# Supplementary material for: The causal role of circulating inflammatory markers in osteoporosis: a bidirectional Mendelian randomized study
Source: Front Immunol. 2024 Jul 18;15:1412298. doi: 10.3389/fimmu.2024.1412298 (PMC11291241; doi:10.3389/fimmu.2024.1412298)
Supplement: Supplementary file 1 [file DataSheet_1.zip › supplementary material/CIM-OSTEOPOROSIS /GCST90274808.csv_forest.pdf]

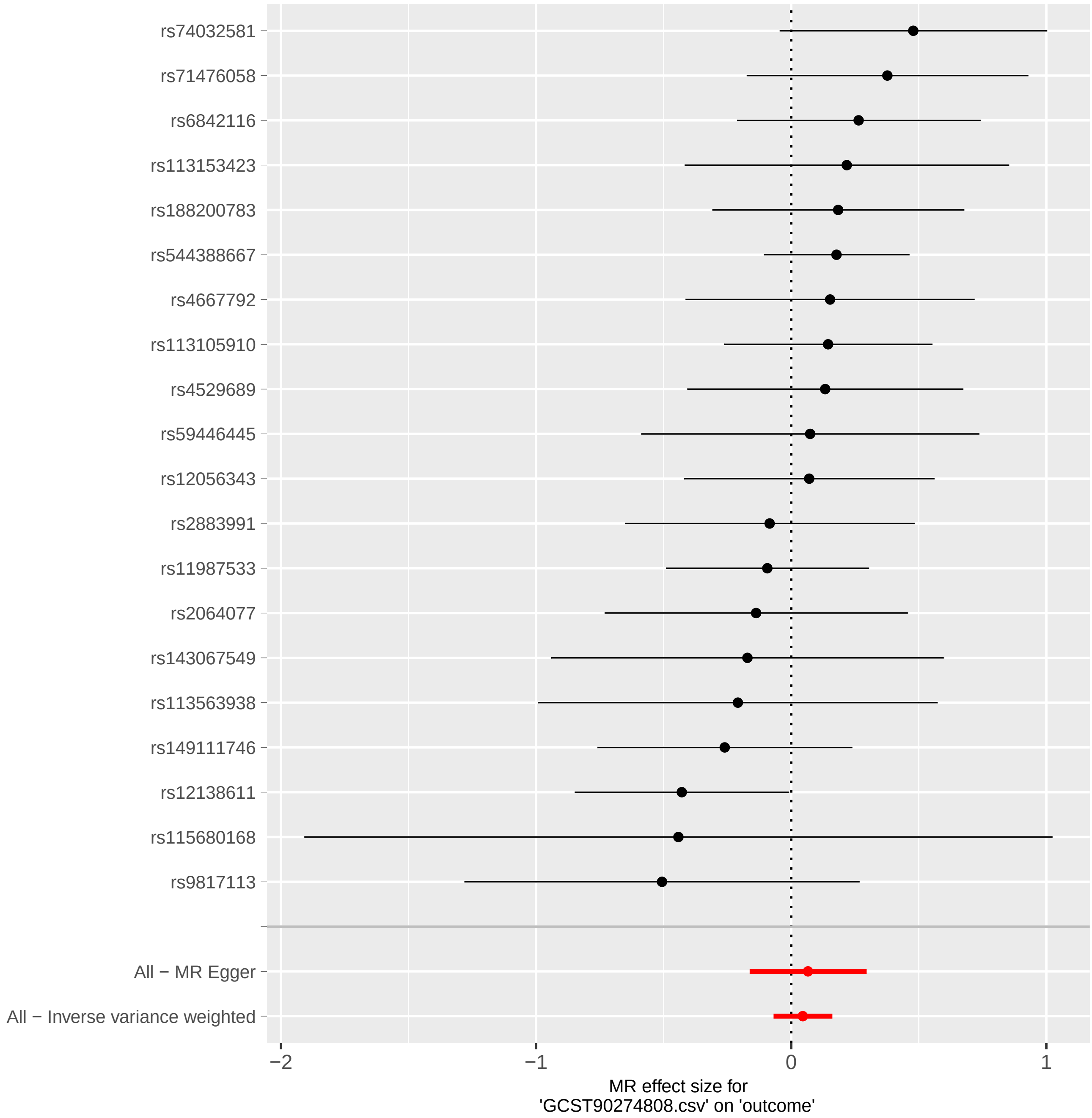
rs74032581
rs71476058
rs6842116
rs113153423
rs188200783
rs544388667
rs4667792
rs113105910
rs4529689
rs59446445
rs12056343
rs2883991
rs11987533
rs2064077
rs143067549
rs113563938
rs149111746
rs12138611
rs115680168
rs9817113
All − MR Egger
All − Inverse variance weighted
−2
−1
0
1
MR effect size for
'GCST90274808.csv' on 'outcome'
